# Measuring adolescents’ level of interest in nature: a promising psychological factor facilitating nature protection

**DOI:** 10.3389/fpsyg.2023.1186557

**Published:** 2023-06-21

**Authors:** Anna-Lena Neurohr, Nadine Pasch, Siegmar Otto, Andrea Möller

**Affiliations:** ^1^Austrian Educational Competence Centre for Biology, University of Vienna, Vienna, Austria; ^2^Biology Education, University of Trier, Trier, Germany; ^3^Department of Sustainable Development and Change, University of Hohenheim, Hohenheim, Germany; ^4^Department of Life Sciences, University of Vienna, Vienna, Austria

**Keywords:** interest in nature, attitude measurements, environmental attitudes, adolescent attitudes, connection with nature, item response theory

## Abstract

Studies indicate that young people are more prepared to engage in pro-environmental behavior if they are interested in nature and recognize it as worthy of protection. However, a reliable instrument to measure adolescents’ interest in nature is still lacking. Therefore, we developed a new metric, the *Scale of Interest in Nature* (SIN). It consists of 18 items, is based on Item-Response-Theory and was validated using the known group approach (*N* = 351 adolescents). Results indicate that adolescents’ interest in nature correlates positively with their connection with nature, their intention to preserve nature and engagement in pro-environmental activities in their free time. Bivariate Pearson correlations between the SIN and the Connectedness to Nature Scale (INS), as well as the Environmental Values model (2-MEV) demonstrated the scale’s construct validity. Hence, the SIN scale provides an economical way to measure adolescents’ interest in nature in research contexts or environmental and sustainability education settings.

## 1. Introduction

With the climate change and biodiversity crises, humanity is facing perhaps the most difficult challenges in its history. Reports about global warming (Al-Ghussain, 2019), sea-level rise (Nerem et al., 2018), permafrost gradation (Colucci and Guglielmin, 2019), glacier melting and retreat (Brighenti et al., 2019) as well as the dramatic loss of biodiversity (Eichenberg et al., 2021) are ubiquitous. Both, the IPBES (2019) as well as the (IPCC, 2021) reports confirm that human behavior and decisions are the main cause of these global effects. Changes are needed in political commitment, institutional frameworks, policies and instruments in order to set clear adaptation goals and define responsibilities and commitments (Pörtner et al., 2022). In addition, individual actions must also change. In regard to this important agenda, researchers worldwide highlight the great potential of the education sector to play an active role in fostering a just climate transition (Anderson, 2012; Lutz et al., 2014; Reimers, 2021; Winter et al., 2022). Otto I. M. et al. (2020) even identified the education system as a “social tipping element” within the “climate tipping elements,” indicating the prominent role of education in helping to activate social dynamics that can stabilize the earth’s climate by 2050 (Otto I. M. et al., 2020; Winter et al., 2022). Here, Education for Sustainable Development (ESD) can respond to the urgent and dramatic challenges the planet faces and offers learners of all ages the knowledge, skills, values and attitudes needed to promote sustainable development and pro-environmental action. ESD is considered a lifelong learning process empowering people to make informed decisions and take individual and collective actions to address national and global challenges—such as climate change or biodiversity loss (UNESCO, 2020). Within this framework, research indicates that different influencing factors, such as environmental knowledge, interests, values, and attitudes, work to form a person’s pro-environmental behavior (Kals et al., 1999; Roczen et al., 2014; Evans et al., 2018; Maurer and Bogner, 2020). These factors include affective, intellectual and behavioral components (Schwartz, 1992; Eagly and Chaiken, 1993; Fishbein, 2010; Kahneman, 2011). Interest, for example can be triggered either by intellectual or affective aspects (Hidi and Renninger, 2006).

Among the various factors influencing environmental behavior, interest in nature seems to play an important role in developing and maintaining environmental knowledge, values and attitudes (Uitto et al., 2011). Studies indicate that young people are more prepared to engage in pro-environmental behavior if they are interested in nature and recognize it as worthy of protection (Kals et al., 1999; Leske and Bögeholz, 2008; Uitto et al., 2011; Cheng and Monroe, 2012). Guiney (2009) demonstrated that the main reasons conservation volunteers actively engage in nature protection were interest in nature at a young age as well as nature-related activities and experiences in adolescence (Kals et al., 1999; Chawla, 2020). Such interest and experience leads to a comprehensive understanding of the natural world and humans’ complex relationship with it, which in turn contributes to environmental awareness and willingness to act (Kals et al., 1999; Bogner, 2007).

Most research on interest in nature concentrates on its intellectual aspects (Kals et al., 1999; Kleespies et al., 2021). However, these intellectual factors (such as environmental knowledge) seem to have little to no influence on environmental behavior (Frick et al., 2004; Abrahamse et al., 2005; Otto and Pensini, 2017). Instead, it is more likely that the motivation to act is triggered by affective or motivational factors, such as personal values, goals, and self-efficacy beliefs, which play a crucial role in driving behavior. These factors can influence individuals’ intentions, decision-making processes, and level of engagement in taking action (e.g., Ajzen, 1985; Deci and Ryan, 2000; Singh et al., 2006). Although interest’s affective factors have not been thoroughly examined regarding their influence on pro-environmental behavior, it seems reasonable to assume that an interest in nature primarily driven by emotions could motivate individuals to actively engage in environmental protection. Therefore, the question arises as to how interest in nature develops over time and how it is related to other factors that influence pro-environmental behavior, such as environmental attitudes, and how it is ultimately affects behavior. Since we already know that most of the foundations for environmentally protective behavior is laid in childhood (Evans et al., 2018; Otto et al., 2019; Chawla, 2020), it can be assumed that formation of an affective interest in nature should also take place at an early age.

This study aims to validate a scale for measuring adolescents’ interest in nature (*Scale of Interest in Nature*, SIN) in terms of its affective aspects—intrinsic, value and emotion-related—in order to provide an age-appropriate assessment instrument. We specifically chose adolescents as the target population because studies show that interest in nature drops with increasing age, especially when puberty hits (e.g., Leske and Bögeholz, 2008). Therefore, we believe it is of great importance to have an instrument at hand that reliably assesses interest in nature in this specific age group in research contexts as well as in environmental and sustainability education settings. Moreover, the newly introduced variable (SIN) could unveil additional attitude traits that affect pro-environmental behavior and enhance comprehension of the interrelations among these attitudes. Furthermore, by looking into existing measures of connectedness to and interest in nature (e.g., Kals et al., 1999; Schultz, 2002; Mayer and Frantz, 2004; Brügger et al., 2011), but also by their conceptualization, we expect interest in nature will most likely establish “only” as a specific but practically meaningful facet of attitude toward or connectedness to nature.

The scale developed for this purpose is based on the interest items by Schiefele et al. (1993) and was adapted to adolescents and to the topic of nature. In developing the items, we included (Markl’s 1989) concept of nature and the biophilic values by Kellert (1993). For validation, we use the known groups approach and compare groups with different frequencies of experiences in nature. Based on former studies (e.g., Guiney, 2009) we hypothesize that adolescents who are more involved in nature-related activities in their free time feel more connected with nature and show a higher affective interest in it.

## 2. Theoretical framework

### 2.1. Environmental attitudes

Researchers emphasize the multidimensional nature of environmental attitudes and assume a framework of intellectual (facts, knowledge or understanding), affective (emotion and feeling) and conative (action and behavior) components (e.g., Fishbein and Ajzen, 1974; Gray, 1985). Educational programs have long provided the foundations of environmental awareness and concern about human impact, which shape the development of environmental behavior (Gigliotti, 1990; Hungerford and Volk, 1990; Bogner, 2004). Researchers have suggested that the most important determinant of behavior is attitude (Eagles and Demare, 1999). The construct of environmental attitudes commonly encompasses multiple components and can be defined as a “collection of beliefs, affect, and behavioral intentions a person holds regarding environmentally related activities or issues” (Schultz et al., 2004, p. 31). However, values might be at the broadest level and are conceptualized as important principles in life (Olson and Zanna, 1993; Schultz et al., 2004). Values function as an organizing system for attitudes and beliefs, and they are viewed as determinants of attitudes. Studies have further emphasized the importance of values in situational and personal interest (Hidi and Renninger, 2006). The term environmental values refers to values that are specifically related to nature or that have been found to correlate with specific environmental attitudes or concerns (Schultz et al., 2004).

Numerous approaches to operationalizing empirical scales have been proposed within the domain of environmental attitudes (Bogner and Wiseman, 2002), which captures attitudes at various levels of specificity, such as attitudes, worldviews, and values. In order to measure adolescents’ environmental attitudes, Bogner and Wiseman (1999, 2002) developed the Environmental Scale (2-MEV). Their first study revealed several subscales of environmental concern, including attitudes, verbal commitment, and actual behavior. Using higher-order factor analysis based on a large pool of items, they developed a model of ecological values (MEV) based upon one’s position on two orthogonal dimensions: Utilization and Preservation. These two values allow a person both to endorse the protection of the environment on a biocentric dimension and to support the utilization of nature on an anthropocentric dimension. The theory of ecological attitudes (EA) posits that people who have strong Preservation (biocentric) attitudes do not necessarily have weak Utilization (anthropocentric) attitudes. This allows individuals to be placed in one of four quadrants rather than on either end of a continuum. The theory explicitly states that Preservation and Utilization are complementary and uncorrelated, not opposing values. Hence, a respondent’s position on one dimension provides no information about his position on the other. At present, the 2-MEV scale’s validity has been independently and repeatedly confirmed by different research groups and has been translated into 33 different languages. Additionally, several researchers have confirmed the bi-dimensional structure of EA, suggesting that Preservation and Utilization are two distinct constructs (Milfont and Duckitt, 2004; Johnson and Manoli, 2010). Therefore, Bogner and Wiseman (2006) offer an age-adjusted item battery for adolescents employing more rigorous psychometric techniques. By measuring environmental attitudes, they expect to obtain a valuable predictor of ecological behavior (Oerke and Bogner, 2013; Maurer and Bogner, 2020; Bogner and Suarez, 2022).

### 2.2. Connectedness with nature

Adolescents are increasingly disconnected from nature, a trend that has significant implications for the preservation of the biosphere (Louv, 2005; Charles et al., 2018; Chawla, 2020). Connectedness with nature is linked to ecological concern and is seen as a lever for societal change toward respect and care for nature (Ives et al., 2017; Otto and Pensini, 2017). This connectedness describes how people form a relationship with elements in the environment (Beery, 2013; Salazar et al., 2021). Numerous studies have identified experiences that create a sense of connection to nature and how this connection is linked to other aspects of life, such as happiness and support for environmental preservation (Tam, 2013; Zylstra et al., 2014; Restall and Conrad, 2015). Spending time in nature and focusing attention on it can enhance this connection (Richardson et al., 2020; Bezeljak et al., 2023).

Furthermore, studies have found that connection to nature is positively associated with pro-environmental behavior and can thus be seen as a significant predictor of it (Mayer and Frantz, 2004; Nisbet et al., 2009; Otto et al., 2014; Roczen et al., 2014; Zelenski et al., 2015; Whitburn et al., 2019, 2020). However, environmental values, attitudes, emotional involvement, social and cultural factors also play roles in explaining environmental behavior (Kollmuss and Agyeman, 2002; Eames et al., 2018). Young people with more access to and experience in nature express a stronger connectedness with it and are more likely to take action to care for nature, even into adulthood (Cheng and Monroe, 2012; Collado et al., 2013; Evans et al., 2018; Barrable and Booth, 2020; Chawla, 2020). Childhood is a crucial period for connecting with nature, but the level of connection tends to decrease to its lowest level in adolescence before slowly rising again in adulthood (Liefländer et al., 2013; Hughes et al., 2019; Richardson et al., 2019).

Regarding the terminology, various terms are employed to describe the concept of connectedness with nature, including affinity, biophilia, ecological self, and nature-relatedness, among others (Beery, 2013). One approach is presented by Schultz et al. (2004), who argue that an individual’s beliefs about the extent to which they are part of the natural environment provides the foundation for the types of concerns they develop, and the types of situations that will motivate them to act. To analyze connectedness with nature, they used the Inclusion of Nature in Self Scale (INS; Schultz, 2002) which examines a person’s relationship with nature. They found that connectedness correlates with biospheric concerns and with self-reported environmental behavior. Therefore, individuals who feel a link between themselves and the natural environment tend to have broader sets of concerns for environmental issues. In contrast, those who feel separate from nature only value nature when it benefits them individually.

### 2.3. Interest in nature

While connection with nature has been interpreted as an environmental attitude (Brügger et al., 2011), personal interest in nature can be seen as the basic factor underlying the development and maintenance of an internal motivation to study nature or act in a pro-environmental way (Uitto and Saloranta, 2010). According to the person-object theory by Krapp (2002), interest represents a specific relationship between a person and an object and encompasses intellectual and affective components (Hidi et al., 2004). Furthermore, he distinguishes between two levels of interest: a situational interest and individual interest (Krapp, 2002). Situational interest describes a motivational state of being interested during an actual activity and is necessary for the development of a sustained, individual interest (Krapp and Prenzel, 2011). Individual interest refers to a person’s dispositional motivational state and is interpreted as a relatively stable tendency to engage with an object of interest without external pressure (Krapp and Prenzel, 2011). Through psychological processes like internalization and identification, the object of interest will be integrated into an individual’s values and feelings and becomes a permanent part of their own identity (Krapp, 2007; Blankenburg and Scheersoi, 2018). Individuals with a well-developed individual interest not only act primarily out of their own motivation but have also developed a persistence to carry on despite failures or negative feelings (Renninger and Hidi, 2002). Therefore, in order to foster a successful development of interest and engagement in an object, individuals need to first identify with the object of interest and to integrate it into their self-concept (Blankenburg and Scheersoi, 2018).

Interest in nature can be considered a type of individual interest, describing the relationship between a person and the object of nature. This relationship is also described in Markl’s (1989) understanding of nature: he advocates for a “biocentric” perspective in which humans recognize that they are part of a larger web of life rather than separate from it. This includes all aspects of the natural world, such as animals, plants and landscapes. In line with this, the biophilia hypothesis argues that humans have an innate interest in life and life-like processes (Wilson, 1984) and need nature for more than just physical survival. Studies support these theories and have shown that an (intellectual) interest in nature has a direct effect on the development of willingness to protect nature and could be therefore a predictor of nature-protective behavior (Langeheine and Lehmann, 1986; Vining and Ebreo, 1992; Kals, 1996; Kals et al., 1999). However, existing studies have concentrated only on intellectual interest in nature, not on its affective character. Even though some researchers suggest that interest is a purely affective construct (Schiefele et al., 1993), it remains unclear what influence an affective interest in nature might have on pro-environmental behavior.

Schiefele et al. (1993) go further in describing affective interest and identify three different components: a value-related, an emotion-related, and an intrinsic component. Whereas the value-related component refers to matters that are personally meaningful to an individual, the emotional component of interest consists of matters that are associated with positive feelings such as pleasure. The intrinsic component involves direct emotional and value attributions to an object or action (Schiefele et al., 1993). Although these components are theoretically well-grounded, they do not separate well analytically in various studies and often load onto a common factor. Taking Schiefele et al.’s (1993) theoretical approach into account, Leske and Bögeholz (2008) examined the influence of interest in nature on the willingness to preserve biodiversity among student in grades 7–12. Their analysis identified the value-related and emotional components as influential predictors. However, they extended the concept of nature used by Kals et al. (1999) to include the terms “biodiversity” and “ecosystems.” Both terms do not merely describe the natural environment, but also emphasize the importance of its conservation and preservation. Thus, the inclusion of these terms in the measurement of interest in nature implies not only a fascination or attraction to nature but also a recognition of the need to protect it for future generations (Miller, 2005). By including these terms, interest in nature does not describe an individual preference anymore, but also carries a sense of responsibility and concern for the well-being of the environment.

However, it remains unclear what influence interest in nature has on pro-environmental behavior and how it is related to other environmental attitudinal variables. Interest in nature could be an additional or rather more direct construct than environmental attitudes and behavior, possibly explaining why students develop greater pro-environmental competence through nature-based environmental education (Otto and Pensini, 2017). Moreover, interest in nature could be a motivation to develop environmental competency similar to a fascination with science, which is an important motivator for engaging in science and supports deeper learning (Otto S. et al., 2020).

Therefore, in this study we aim to develop and validate a comprehensive self-report instrument that accurately assesses adolescents’ interest in nature: the Scale of Interest in Nature (SIN). We want to demonstrate the measure’s reliability by confirming the internal consistency of the construct and by confirming unidimensionality. Assuming that attitudes toward nature and attitudes toward environmental protection represent different dimensions (Kaiser et al., 2013), we predict interest in nature to be on the same dimension as the inclusion of nature in self (Schultz, 2002), while preservation (Bogner and Wiseman, 2006) should be on a separate dimension. To ensure the construct validity of SIN, a known-group comparison was used. Using SIN, we hope to provide an assessment tool for adolescents’ interest in nature, which can be helpful in research settings as well as in formal or informal education for sustainable development.

## 3. Methods

### 3.1. Participants and procedures

In 2017 and 2018, a sample of 351 adolescents (average age = 12.58 years, SD: 1.58, range: 10–15; 41.9% girls) were selected from different interest groups in Germany (Rhineland-Palatinate, North Rhine-Westphalia, and Saarland) and Austria (Vienna and Lower Austria). We employed the method of comparing of known groups as a validation criterion for the scale, as this approach has been previously utilized to assess pro-environmental and pro-social behavior (e.g., Neaman et al., 2021; Otto et al., 2021). Known-groups validity is demonstrated when a questionnaire can discriminate between two groups known to differ on the variable of interest. Based on previous research, we chose environmental organizations, humanitarian organizations, and sport clubs (i.e., Scarborough, 2013; Otto et al., 2021). This prior research has shown that connectedness to nature is higher in students enrolled in environmental studies compared to students enrolled in other courses, in park management students compared to sports management students, and in members of environmentalist groups compared to humanitarian groups. As interest in nature is theoretically related to attitudes and general ecological behavior, we expect that members of environmental groups spend more time outside, feel more responsible for nature and show a higher interest in nature and the environment than members of other interest groups. To test this hypothesis, we linked participants’ interest in nature with their free time activities. Therefore, we divided the sample into three different interest groups:

1. Members of Sports Clubs (*N* = 133, e.g., football, handball or track and field), who were expected to express no specific interest in nature or nature protection. This group has chosen its hobby because of the physical activity involved. In personal conversations, the adolescents claimed that they chose football, for example, because of the sport itself and not as a way to spend more time outside;
2. Members of Socially Engaged Associations (*N* = 112, e.g., the Red Cross or other welfare organizations), who are engaged in voluntary work, but not with regard to nature or the environment; and
3. Active Members in Various Groups or Organizations for Nature Protection (*N* = 106, such as participants in Junior Ranger Programs or the youth associations of national nature conservation groups), who engage with protecting nature in their free time and also conduct nature protection activities as private individuals. To ensure that all participating groups are really focused on nature and its protection, we accompanied and observed them as they conducted weekly nature conservation activities. These activities include hanging birdhouses, building bat shelters, and planting trees.

We identified sport clubs, humanitarian and environmental organizations in the respective regions via the internet and then made individual appointments with the different groups to collect the data. The selected organizations were the same in Germany and Austria (e.g., the German and the Austrian Red Cross) in order to ensure that the organizations had equivalent aims. Participants completed the questionnaire at their regular group meetings during their free time. To prevent overlap in interest groups, the questionnaires asked about participation in other organizations. Only 4.08% of participants demonstrated double engagement. Of the 360 participants who were provided with the paper-and pencil questionnaire, 351 completed it (response rate: 97.5%).

### 3.2. Measures

The 2 Major Environmental Values model scale (2-MEV, Wiseman and Bogner, 2003) comprises 18 statements addressing the constructs of Preservation and Utilization (see Supplementary material 1). The Preservation measure was derived from students’ responses to 9 items, such as “I take care to save water and electricity.” Utilization was also measured with 9 items, such as “Humans are more important than other living beings (e.g., animals and plants).” Participants gave their responses on a 5-point Likert scale from 0 (strongly disagree) to 4 (strongly agree). Conventional principal factors’ extraction with varimax rotation confirmed the proposed two-factor solution. In total, the two-factor model accounted for 40.42% of the common variance in the data. Scores on the individual levels were calculated in accordance with convention as mean values for the 18 items using a Rasch-scale calibration. Although the 2-MEV was originally subject to a factor analysis, we believe a Rasch-based analysis provides numerous advantages. Even if participants do not respond to the exact same set of items, they can still be quantitatively compared as long as the scales have some core overlapping items (Kaiser et al., 2018). Because the estimation procedure is based on a maximum likelihood approach, attitude estimates can be attained even with incomplete data sets that contain missing values (Baierl et al., 2022). Further, Kaiser et al. (2018) propose that the Rasch model can assure a specific objectivity by ordering indicators transitively with respect to their difficulty. The type of indicator is not a defining feature of environmental attitudes, but the “numerical relations of magnitudes of psychological attributes incorporated in people” is (Kaiser et al., 2018, p. 141). The results of the analyses are in line with previous studies (Bogner and Wiseman, 2006) and revealed acceptable internal consistencies for both Preservation (*α* = 0.79) and Utilization (*α* =0.74).

Inclusion of nature in self (INS) is the second scale used in this study. The INS is widely used as an assessment of nature connectedness and is based on only one item (Schultz, 2002). By means of a series of seven differentially overlapping circles (labeled “self” and “nature”), participants could choose the one that best described how connected they felt with nature. Compared to other multiple-item scales, the INS has been found to be very accurate for measuring individual connectedness with nature and correlates well with other connection with nature instruments (Brügger et al., 2011). Scores range from 0 to 6, with the circle with the least overlap receiving a score of 0 (complete separation from nature) and the most overlapping circle receiving a score of 6 (complete connection to nature) (see Schultz, 2002). Since this measure is a single-item measure, its reliability could not be estimated with our data. Nevertheless, its 4-week test-retest reliability is reported to be rtt = 0.84 (Schultz et al., 2004).

### 3.3. Development of the scale of interest in nature

Our newly developed scale of interest in nature (SIN) is a composite of 18 interest items (see Table 1), which were adapted from Schiefele et al. (1993). The item set was reduced statistically from initial 43 items via item response theory (see Supplementary material 2 for the initial set of 43 items). In contrast to other research groups (Prenzel et al., 1986; Kleespies et al., 2021), Schiefele et al. (1993) present a distinct perspective on the nature of interest. They conceptualize interest as a purely affective construct, separated from any intellectual or knowledge-related components. They propose indeed three components of affective interest: an emotion-related component, a value-related component, and an intrinsic component.

**Table 1:** Item fit values of all the 18 items for the Scale of Interest in Nature (SIN) of the pilot and of the final study, adapted from Schiefele et al. (1993).

POI dimension	Original item (Schiefele et al.), translated	Items in the final questionnaire		MNSQ pilot study (*N* = 256)	MNSQ final study (*N* = 351)
		#	Item		
Emotion-related valences	Working with the subject matter and problems of my major is not really among my favorite activities	IN 27	I enjoy discovering nature with my friends more than playing computer games or video games with them	1.16	0.94
	I do not like to talk much about the subject matter related to my studies	IN 5i	Plants are boring	1.15	0.98
		IN 37	It is exciting to examine bees or other insects with a magnifying glass	0.88	0.93
	I prefer to talk about my hobbies rather than about my major	IN 13i	I prefer talking about new movies and music, rather than animals	0.83	1.11
	When I am in a library or bookstore, I like to browse through magazines or books with topics related to my major	IN 15	In libraries, I like reading nature books (for example on animals or plants)	0.64	0.91
	A reference book as a birthday present would not give me any particular pleasure	IN 3	In my opinion, documentaries and movies on nature are interesting	0.83	1.00
		IN 16	I would be happy to receive a calendar with nature pictures (for example animals or landscapes) for my birthday	0.92	0.95
	Many areas within my major do not mean anything to me	IN 17i	I do not really mind the fact that humans destroy nature	0.70	1.10
Value-related valences	It was of great personal importance to be to be able to study this particular subject	IN 24	It is important to me to know the names of local animals and plants	0.67	0.92
		IN 26	Personally, I find it important to know the role of humans in nature	0.68	1.06
	To be absolutely honest, I feel sometimes rather indifferent toward my major	IN 22i	If I am being completely honest, I do not care about animals and plants at all	1.05	0.99
	Compared to other things that are of great importance to me (e.g., hobbies, social life), my studies are of markedly less significance to me	IN 7i	I have no personal interest in what happens in nature	0.60	1.12
	I cannot imagine pursuing the content of my studies as a hobby*	IN 28	I could imagine collecting feathers, leaves or other things as a hobby	1.10	0.86
Intrinsic orientation	If I had enough time, I would work more intensively with certain aspects of my studies, even if they had nothing to do with any course requirements	IN 21	In my spare time I take pictures of flowers, animals and landscapes	1.08	1.12
	In my free time, I am unwilling to deal with problems in my field of study*	IN 34	In my spare time I examine plants and conduct small experiments with them (e.g., poking them gently, blowing at them)	1.11	0.96
		IN 30	In my spare time I participate in projects on preserving nature	0.86	1.06
	Even before coming to college I voluntarily spent time thinking about the subject matter of my major (e.g., read books, went to lectures, had conversation with others)	IN 36	Outside of school, I seek out information about animals and plants (for example on the internet or in books)	0.81	0.70
	I chose my major primarily because of the interesting subject matter involved	IN 42	I purposely chose hobbies that allow me to spend a lot of time in nature (e.g., riding, fishing, geocaching)	1.25	1.16

*own translation due to missing translation by the author, “*i*” indicates an inverted item.

The emotion-related component involves positive emotional experiences and thoughts associated with the object of interest. The value-related component pertains to the personal significance or attributions linked to the object. Finally, the intrinsic component, which Schiefele et al. (1993) consider the most crucial, refers to self-intentionality. It represents a person’s engagement with the object for its own sake, driven by internal motivation rather than external rewards or incentives. By emphasizing the intrinsic component, interest is distinguished from other forms of motivation that are externally driven. It describes then a self-determined and autonomous interest that arises from the inherent qualities of the object itself. This approach is consistent with former studies, which have suggested that intellectual variables have little or no effect on environmental behavior (Frick et al., 2004; Abrahamse et al., 2005; Barth et al., 2012; Otto and Pensini, 2017; Knutti, 2019).

To incorporate the idea of nature into our items, we utilized both Markl’s (1989) concept of nature and Kellert’s (1993) biophilic values. Both researchers emphasize the importance of understanding and valuing nature for the sake of environmental protection. Markl (1989) focuses more on the emotional aspects of human-nature relationships, underlining the importance of experiencing wonder in nature as part of one’s spirituality. He suggests that humans should strive for a harmonious relationship with nature based on respect and humility. Similarly, Kellert’s concept of biophilia emphasizes the innate human connection to nature and the importance of maintaining the connection for our well-being and the preservation of the natural world. However, unlike other studies (Kals et al., 1999; Leske and Bögeholz, 2008), we did not take into account resources, biodiversity and ecosystem in our items. These aspects encompass the idea of environmental preservation and thus express motivation rather than interest. We found support in this decision in Kaiser et al. (2013), who found that attitude toward nature and attitude toward nature protection represent two separate constructs. The items were formulated using age-appropriate language and considering the reality of adolescents’ lives.

We measured interest in nature with 18 self-reported items on a 5-point Likert scale (0 = strongly disagree to 4 = strongly agree). To control for response style bias, the scale included 5 inverse items, which we recoded afterwards. In line with Kaiser and Wilson (2004), the answers to the polytomous items were recoded into a dichotomous format by collapsing strongly disagree, disagree and partially agree as indicators for a lack of interest in nature. The responses agree and strongly agree were combined to indicate an interest in nature. This dichotomization practice is an established precaution to guard against excessive measurement error, particularly in attitude research (DeCoster et al., 2009; for supporting evidence, see, e.g., Kaiser and Wilson, 2004; Byrka et al., 2016). For all items, “Not applicable” was an alternative response when an answer was not possible: such responses were treated as missing values. Rasch model calibrations and therefore person score estimations can be gained even with incomplete data records, as this estimation is based on a maximum probability procedure (Embretson and Reise, 2000; Linacre, 2002; Kaiser et al., 2007; Boone et al., 2014). In addition, we collected socioeconomic data from the adolescents such as age, gender, grade, type of school and the native language.

Using the methodology of parceling (Little et al., 2002), the items of the SIN scale were reduced statistically from 43 to 18 items via item response theory (see Supplementary material 2 for the 43 initial items). This method involves grouping multiple observed variables together into smaller parcels, which are then used as indicators of latent variables. It is mostly used in structural equation modeling (SEM) to create composite variables or parcels from observed indicators. Parceling aims to improve the efficiency and stability of the analysis by reducing the number of observed variables and increasing the reliability of the parcels. We reviewed Item Infits (MS Infit <1.3; Wright et al., 1994) and the difficulty distribution on a Wright map (person-item map). A Wright map is a graphical representation of a Rasch model that visually displays the performance of items and persons on a single scale, demonstrating the fit between items and persons and providing information about any patterns or anomalies in the data (Linacre, 2021). It is a useful tool for evaluating the performance of a questionnaire and can be used to identify problematic items. Items that are on the same level on the Wright Map cover the same degree of interest in nature. In order to reduce the number of items and ensure that all levels of interest were covered, we removed items that were located on the same level in the Wright Map. As a result of this reduction, we lessened disturbance variants, minimized scattering and errors, and enabled a normal distribution (Bandalos and Finney, 2001). This adaptation was justified by two pilot studies (first pilot: *N* = 79; second pilot: *N* = 177) in grades 5–8 (10–15 years old) in German schools (Rhineland-Palatinate) using the original scale (item rel.: 0.96, person-rel.: 0.92 and MNSQ Infit: 0.62–1.45). The indices for item reliability, person reliability, construct validity, normal distribution and model fit were checked, which attested to the test quality (e.g., Linacre, 2009; Boone et al., 2014). Mean-Infit MS-Values (MNSQ) up to 1.3 suggest a reasonable fit of the data to the model (Wright et al., 1994).

### 3.4. Statistical analysis

The Rasch model was used to analyze the measurement data (Linacre, 2002). This model can obtain specifically objective (i.e., item and person-independent) test results. Specific objectivity in this context means that two persons can be quantitatively compared with each other regarding a latent attribute (e.g., environmental attitudes) even if different measurement instruments have been used to assess the attribute (for more details, see Kaiser et al., 2018). Therefore, the specific objectivity can be seen as a formal validation criterion. To test if the developed test items fit the Rasch model, we analyzed the model fit indices. To further evaluate the test quality, reliability indices such as person reliability, person separation and item reliability were also checked (Bond et al., 2020). Person separation was used to classify individuals and ensure that the instrument can effectively differentiate between high and low performers. Item separation was employed to validate the item difficulty hierarchy, which demonstrated the construct validity of the instrument (Boone et al., 2014; Boone and Staver, 2020). Malec et al. (2007) suggests the following critical values: item reliability of 0.90, person reliability of 0.80, person separation of 2.0, and item separation of 4.0. Due to the relatively large sample size, we relied on the mean square values (MS Infits) in the assessment of item fit, where values lower than 1.3 indicate an acceptable fit (O'Connor et al., 2016; Linacre, 2021).

The discriminant and convergent construct validity of the newly developed scale were evaluated with two well-established environmental attitude instruments (INS: Schultz, 2002; 2-MEV: Bogner and Wiseman, 1999). To demonstrate that the instrument is unidimensional and internally consistent, we conducted a principal-axis factor (PAF) analysis based on the theoretical factors established in the instrument design process. Analyses were done with a varimax rotation. The inclusion of nature in self scale (INS) is designed to measure the degree to which people include nature in their self-concept (Schultz, 2001). Since both the INS scale and interest in nature measure individuals’ psychological connection and affinity with the natural world, comparing the scores on these two measures can provide evidence of convergent validity. To show discriminant validity, the Preservation items by Bogner and Wiseman (1999) were used. Kaiser et al. (2013) postulates a two-dimensional attitude model, which distinguishes between appreciation for nature (which we measure with SIN) and appreciation for environmental protection (which can be called preservation).

To ensure construct validity, we compared groups with different frequencies of experiences in nature (known-groups) using an analysis of variance (ANOVA). Following the known groups approach, we assume that members of an environmental organization, respectively, show a higher-than-average pro-environmental motivation (Otto et al., 2021). Data analysis was conducted with the Rasch software Winsteps (Linacre, 2015) and SPSS 26 for further calculations.

## 4. Results

The present findings are reported in two parts. First, we describe the calibration of the proposed SIN scale using the partial-credit Rasch model in order to evaluate the construct validity with the 2-MEV (Wiseman and Bogner, 2003) and INS (Schultz, 2002). Here, we present the test quality indices. Second, we also present the comparison of the known groups to demonstrate the construct validation of the newly developed scale.

### 4.1. Psychometric quality of the scale of interest in nature

To assess the construct validity of the three instruments used, we performed a principal-axis factor analysis (PAF) with a varimax rotation, extracting three factors. The objective was to confirm that the items representing the constructs of Interest in Nature, Preservation, and Utilization loaded significantly on their respective factors. The PAF was employed to establish the unidimensionality and internal consistency of each of the three above mentioned constructs, based on the underlying theoretical framework. The Kaiser-Meyer-Olkin measure of sampling adequacy was 0.81, above the commonly recommended value of 0.5, and Bartlett’s test of sphericity was significant (*p* < 0.001), indicating that correlations between items were sufficiently large to perform a PAF. Examination of Kaiser’s criteria and the scree plot yielded empirical justification for retaining three factors with eigenvalues exceeding 1, which accounted for 31.94% of the total variance.

Based on the theoretical framework, we assumed three factors to be examined: Interest in Nature, Preservation and Utilization. The analyses indicated that most of the items’ factor loadings resembled the theoretical structure (see Table 2). Some unexpected cross-loadings were found between the value-related interest in nature items and the preservation items. However, this can be explained with reference to the theoretical derivation of the two constructs. Only if a person considers nature to be valuable will he or she also commit themselves to it and protect it. For this reason, the Preservation construct already contains value-related tendencies. If the SIN is used together with the 2-MEV, the 5 value-related items could be cut (Table 1), since these are already reflected in the preservation items (in the following “SIN re” refers to the reduced version of the scale consisting of 13 items). The internal consistency of the reduced SIN scale and bivariate correlations can be found in Table 3 (see variable SIN re). However, since the aim was to create a stand-alone instrument, these items remain included in the further analyses, and the results presented below pertain to the entire SIN scale (with 18 items).

**Table 2:** Standardized loadings on the dimensions emotion-related, value-related and intrinsic interest in nature (SIN) as well as Preservation (PRE) and Utilization (UTL) for the sample of adolescents (10–15 years old) (*N* = 351).

Item		SIN	PRE	UTL
**Emotion-related**				
IN3^a^		0.52		
IN5i^a^		0.38		
IN13i^a^		0.47		
IN15^a^		0.55		
IN16^a^		0.43		
IN17i^a^			0.43	
IN27^a^		0.38	0.39	
IN37^a^		0.55		
Value-related				
IN7i^a^			0.34	
IN22i^a^			0.42	−0.31
IN24^a^		0.49		
IN26^a^		0.35	0.39	
IN28^a^		0.68		
Intrinsic				
IN21^a^		0.46		
IN30^a^		0.47		
IN34^a^		0.53		
IN36^a^		0.74		
IN42^a^		0.39		
UTL1^b^				0.49
UTL2^b^				0.49
UTL3^b^				0.41
UTL4^b^			−0.33	0.50
UTL5^b^				0.48
UTL6^b^				0.42
UTL7^b^			−0.34	0.63
UTL9^b^			−0.44	0.46
UTL10^b^				0.55
PRE1^b^			0.55	
PRE2^b^		0.36	0.62	
PRE3^b^			0.49	
PRE4^b^				−0.50
PRE5^b^		0.31	0.51	
PRE7^b^			0.65	
PRE8^b^		0.58	0.46	
PRE9^b^			0.48	
PRE10^b^				−0.56

Inverse items are marked with an *i* (for inverted); loadings under 0.3 were suppressed.

Item (s) source:

a Modified from Schiefele et al. (1993).

b Modified from Bogner and Wiseman (2006).

**Table 3:** Descriptive statistics and bivariate correlations of attitudes toward nature (Preservation and Utilization), Inclusion of nature in self (INS) and interest in nature (total and reduced).

	*M*	SD	*N*	SIN	SIN re	PRE	UTL	INS
Interest in nature (SIN)	−0.34	1.68	351	0.99	0.98	0.16	−0.29	0.61
Interest in nature (SIN, reduced)	−0.66	1.76	351	0.96**	0.99	0.15	−0.20	0.61
Preservation (PRE)	0.63	0.80	351	0.16**	0.15**	0.96	−0.14	0.18
Utilization (UTL)	−0.61	0.91	351	- 0.28**	−0.20**	- 0.14*	0.98	−0.23
Inclusion of nature in self (INS)	4.41	1.53	335	0.56**	0.56**	0.16**	- 0.21**	0.84

The reduced version of the SIN does not include the 5 value-related items and thus comprises only 13 items.

Using a Rasch analysis, we found that all item statistics lay within a valid range and indicated a good test quality for the developed SIN scale (Item-Rel.: 0.99, MNSQ Infit mean: 0.99). All 18 items fitted the model prediction with reasonable MS-values between 0.70 and 1.16. None of the items fell outside the tolerable range of fit (i.e., MS < 1.3; *cf.* Wright et al., 1994; Linacre, 2009; Boone et al., 2014). The Rasch-model based person reliability of our developed scale was thus also found to be good with person rel. = 0.81 (*N* = 351).

The convergent validity of interest in nature was derived from the pattern of correlations between interest in nature and inclusion of nature in self. In addition to its intellectual dimension, inclusion of nature in self contains an affective dimension which describes the feeling of a connection to and desire to care for nature (Schultz, 2002). Thus, an emotional affinity can arise between a person and nature (Kals et al., 1999). These emotion-and value-related aspects can also be found in the construct of interest in nature. As expected, the measurement-error-attenuation-corrected Pearson correlation between the two instruments showed that they substantially overlap (i.e., r_corr_ = 0.61; see Table 3).

Discriminant validity was inferred by the correlations between the interest in nature and the two environmental attitudes - Preservation and Utilization. Kaiser et al. (2013) present a two-dimensional attitude model, distinguishing between appreciation for environmental protection and appreciation for nature. We therefore assume that interest in nature reflects a different dimension than Preservation and Utilization. This idea is also supported by the preceding PAF, where items from these scales loaded on different factors. Both measures exhibit only a small to moderate correlation with interest in nature (0.16 < r_corr_ < −0.29), which shows the measured constructs to be unrelated or only slightly related.

### 4.2. Known groups comparison

The known-group comparison is based on the assumption that participants who are less involved with nature or its protection in their free time will also show little interest in nature. To test this hypothesis, we linked participants’ interest in nature with their free time activities. Our results indicated that this was the case. Specifically, we defined three different interest groups: a sports group, a socially engaged group and a group demonstrating an active commitment to nature and environmental protection.

**Figure 1:**
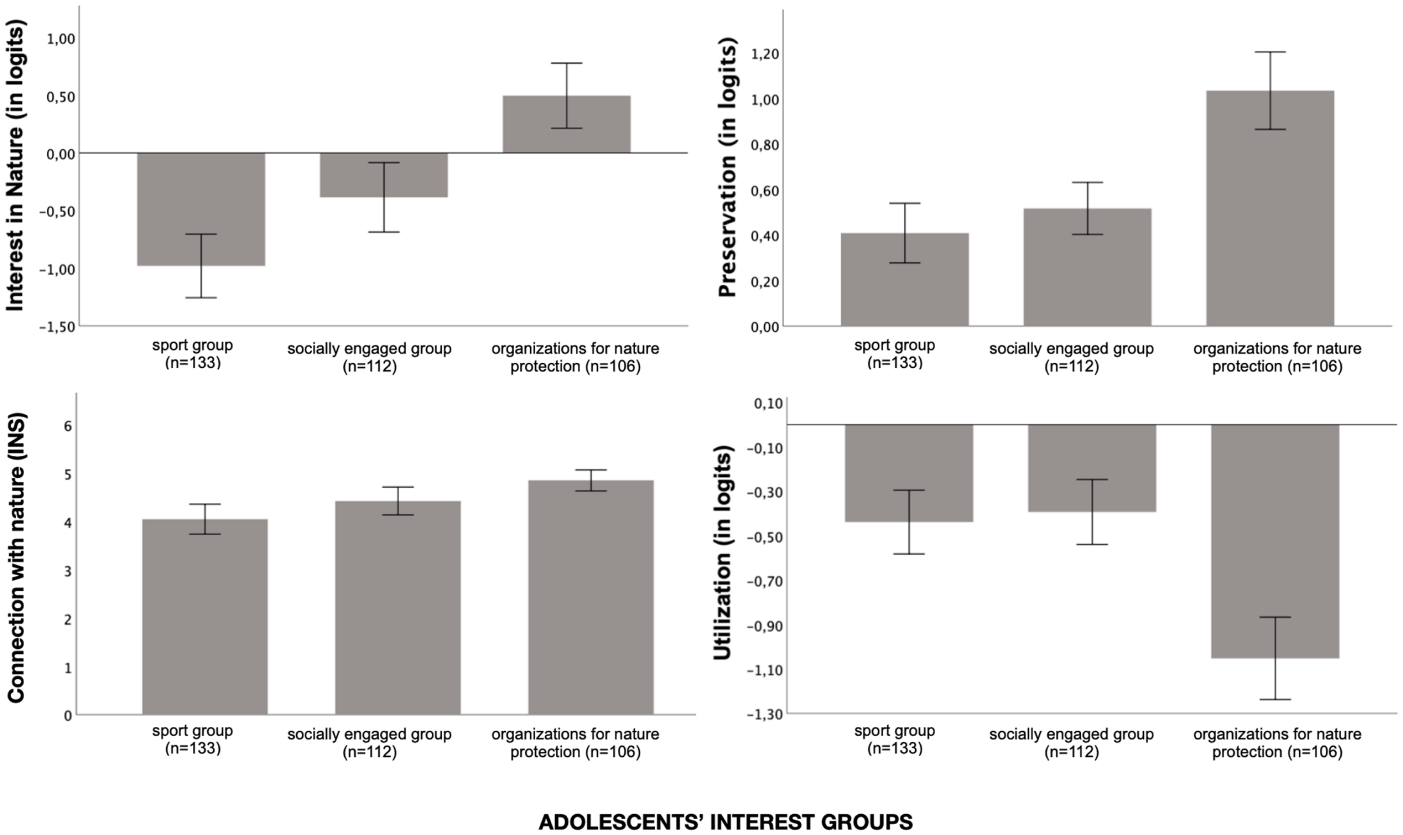
Differences in self-reported Interest in Nature (SIN), Preservation (PRE), and Utilization (UTL), as part of the 2-MEV, and self-reported Connection with nature (measured by the Inclusion of Nature in Self scale, INS) among the three interest groups: sport group, socially engaged group and group involved in organizations for nature protection; mean values and 95% confidence intervals.

A first analysis of variance (ANOVA) indicated a significant difference between groups in interest in nature: *F*(2, 348) = 26.28, *p* < 0.001, *d* = 0.94 (see Figure 1). For theoretical reasons, we expected that the assumed stronger attitudes in favor of environmental protection among members of a nature conservation organization would be linked to a higher interest in nature. Accordingly, we discovered that participants in nature conservation groups held a more pronounced interest in nature (*M* = 0.50, SD = 1.47) than participants in socially engaged groups (*M* = −0.38, SD = 1.61) or sports groups (*M* = −0.98, SD = 1.61). To examine the construct validity of the interest in nature scale, we compared the three different interest groups. *Post hoc* comparisons using t-tests with Bonferroni correction indicated highly significant differences (sports group and socially engaged group, *t*(243) = −2.89, *p* = 0.01, *d* = −0.37; sports group and nature protection organization, *t*(237) = −7.33, *p* < 0.001, *d* = −0.96; socially engaged group and nature protection organization, *t*(216) = −4.22, *p* < 0.001, *d* = −0.58). Overall, we discovered a continuum of interest from the sports group to the social group to the nature group.

Additionally, we were able to show that the three interest groups have different preferences for environmental preservation (Figure 1). Nature group participants’ preservation level (*M* = 1.03, SD = 0.88) significantly surpassed that of both the socially engaged group (*M* = 0.52, SD = 0.61, *t*(216) = −5.06, *p* < 0.001, *d* = −0.67) and the sports group (*M* = 0.41, SD = 0.76, *t*(237) = −5.87, *p* < 0.001, *d* = −0.76). By contrast, there was no evidence for a significant difference between sports group participants and participants in socially engaged groups (*t*(243) = −1.21, *p* = 0.23).

In terms of Connection to Nature and Utilization, no significant difference emerged between participants in sport groups (INS: *M* = 4.05, SD = 1.74; UTL: *M* = −0.44, SD = 0.84) and socially engaged groups (INS: *M* = 4.42, SD = 1.52; UTL: *M* = −0.39, SD = 0.78). However, if we compare participants in both the sport group and the socially engaged group with environmental organization members (INS: *M* = 4.85, SD = 1.10; UTL: *M* = −1.05, SD = 0.96), significant differences can be seen (*p* < 0.001, see Figure 1). Cohen’s d values show small effects (0.158 < *d* < 0.353).

## 5. Discussion

Interest in nature seems to have an influence on willingness to preserve nature (Kals et al., 1999), but it remains unclear how it is related to environmental attitudes and behavior. Interest in nature might be a construct and motive explaining why adolescents develop greater pro-environmental behavior (Otto and Pensini, 2017; Otto S. et al., 2020). To analyze these relationships better, a comprehensive instrument to measure interest in nature is needed. To this end, the aim of this study was to develop a valid instrument to assess adolescents’ interest in nature.

We therefore developed an age-appropriate scale for measuring adolescents’ interest in nature, the SIN. Using Wright Maps and already established scales for measuring environmental attitudes we were able to establish construct validity (convergent and divergent). In addition, the results from known group comparisons support the construct validity of our instrument. Unless stated otherwise, the results presented for the SIN scale pertain to the overall scale consisting of 18 items.

Both the principal-axis factor analysis, and the Rasch-model analysis support the assertion that the SIN has a one-factor structure. The different items seem to measure different parts of one latent construct. As in the underlying scale (Schiefele et al., 1993), this study confirmed that the theoretically postulated interest components—namely emotion-related and value-related valences as well as the intrinsic character of interest—cannot be conceived as independent (orthogonal) factors. Even though the interest components represent covarying aspects of the interest construct, we nevertheless consider their analytic distinction useful and necessary (Krapp, 2002, 2007).

The factor analysis confirms that the constructs Preservation and Utilization do not load onto the same factor as the SIN scale. These data align with Kaiser et al. (2013), who find that attitude toward nature and attitude toward environmental protection are two separate but correlated constructs. However, the factor analysis of the SIN and 2-MEV items together showed that some value-related items not only loaded onto the respective factor of SIN, but also cross-loaded on the Preservation factor of the 2-MEV. The 2-MEV measures environmental values and includes various subscales of environmental concern. Thus, it is to be expected that the items also contain a value-related tendency, which is thus related to some of the SIN items. In any case, SIN provides more detailed information on the emotion-related and intrinsic components of interest in nature.

Furthermore, the analysis confirmed that the newly developed measure is an instrument with reasonable psychometric quality, namely good item fit, reliability, and internal consistency. In terms of convergent validity, the SIN correlated strongly with the INS scale (inclusion of nature in self, Schultz, 2002) and thus should be seen as a more specific dimension within the construct of nature connectedness, but with high practical usefulness. While connection to nature encompasses a broader concept that includes emotional, cognitive, and behavioral components, interest in nature specifically focuses on the level of attraction, curiosity, and engagement individuals have toward the natural world. Regarding discriminant validity, we found low correlations between the SIN scale and the environmental attitude scales Preservation and Utilization (MEV-2 model, Bogner and Wiseman, 2006). By excluding items related to interest in nature preservation from our instrument, our scale will be fully distinct from these aspects and the 2-MEV. Unlike previous studies (e.g., Kals et al., 1999; Leske and Bögeholz, 2008), we did not include measures referencing resources, ecosystem and biodiversity in our item set.

The results show that connection to nature is not only related to interest in nature, but also slightly related to environmental attitudes, in this case Preservation and Utilization. Thus, even though the two scales (INS and 2-MEV) represent two different constructs, the data shows a relationship between them. This suggests that the degree to which a person associates themselves with nature is related to their attitude toward nature protection. Therefore, a person with a stronger connection to nature is more concerned about environmental issues. This is consistent with previous studies showing that nature connectedness and environmental attitudes are substantially related (Schultz et al., 2004; Sellmann and Bogner, 2013; Otto and Pensini, 2017). Moreover, Roczen et al. (2014) integrated nature connectedness into their environmental competence model and considered it an important factor influencing individual environmental behavior. In our study, the strongest level of connection to nature was found among members of environmental organizations. We assume that frequent (positive) experiences with nature increased their individual connection to nature. Even though positive changes in connection are already apparent after one-day environmental education programs, a long-term connection to nature can only be achieved after longer, repeated nature experiences (Stern et al., 2008; Kossack and Bogner, 2012; Sellmann and Bogner, 2013; Möller, 2021). We also identify a trend across the various interest groups in interest in nature. We find the strongest connection to nature in the nature groups, which often spend longer periods of time in nature and deal with topics related to nature conservation (i.e., planting trees, installing nest boxes for birds etc.).

The SIN measure discriminates well between the three interest groups. In the present sample, the higher the SIN, the more likely that one is a member of an environmental organization, that aims to motivate and guide adolescents toward more environmentally friendly behavior. Within the study samples, drawn in Germany and Austria, we also found that adolescents’ self-reported interest in nature was significantly related to the kind of activities they engage in in their free time. We found that members of environmental organizations reported a stronger interest in nature than did members of sports groups or socially engaged groups. We also found the same effect for the connection to nature (INS) and the environmental attitude scales Preservation and Utilization of nature (2-MEV). Our hypothesis suggests that frequent interactions with nature enhance the level of personal engagement among adolescents actively involved in environmental organizations, ultimately leading to a greater interest in nature. Only those who identify with the object of interest will develop an individual interest (Krapp, 2002). The result of individual interest is an experience of positive emotions, increased appreciation, and a consolidation of knowledge about the subject matter (Renninger and Hidi, 2002). We assume that adolescents involved in environmental organizations have developed an individual interest in environmental issues due to their positive experiences in nature and that they have integrated nature as an important aspect of their identity. They have rather low values regarding utilization of nature in this study, which indicates that they do not place humans above nature and therefore do not believe that people should exploit nature for their own needs, which also fits well with their high values on the Preservation-scale.

The results of the known-group comparison align with previous studies highlighting the significance of prosocial propensity in the ecological domain (e.g., Otto et al., 2021). Prosocial propensity refers to an individual’s inclination to engage in actions that benefit others or society as a whole. Within the ecological domain, this propensity is reflected in a willingness to participate in behaviors that protect and conserve the environment, such as recycling, reducing energy consumption, and supporting conservation initiatives (Neaman et al., 2021). By cultivating a strong prosocial propensity, individuals are more likely to take actions that benefit the environment, promote sustainability, and yield positive outcomes such as a reduced ecological footprint, increased involvement in environmental initiatives, and the formation of collective efforts to address environmental challenges. Prosocial propensity in the ecological domain plays a crucial role in fostering a sense of responsibility, empathy, and collective action toward environmental protection, ultimately contributing to a more sustainable and harmonious relationship between humans and the natural world (Otto et al., 2021). In our study, adolescents engaged in social activities also demonstrate a stronger connection to nature and a higher level of interest in the natural world. While their environmental attitudes may not be as pronounced as those of adolescents involved in environmental organizations, they still fall within a higher range compared to members of sports groups.

A limitation of our study is that pro-environmental behaviors and time spent in nature were not directly assessed, but rather assumed based on membership in environmental organizations. However, the examined groups were carefully selected according to strict criteria. It was crucial for us that the meetings of the environmental groups took place outdoors in natural settings and involved activities related to nature and environmental conservation, such as hanging bird houses or planting trees. This approach aimed to ensure that the adolescents not only regularly spent time in nature but also actively participated in nature conservation activities. To ensure the groups’ suitability and adherence to these criteria, we accompanied them on-site. However, in order to make statistically robust statements about the causal relationship between engagement in various leisure activities, time spent in nature, and pro-environmental behavior, future studies should measure these constructs using self-reported variables, such as the General Ecological Behavior scale (Kaiser and Wilson, 2004). Based on the present study, we cannot make any causal statement. This could be addressed in future studies.

Another limitation is the composition of the three interest groups. Not only do they differ in sample size, but they were also collected at different locations. This was due to the challenge of finding enough adolescents in the respective groups willing to participate in the study. In addition, in this age group, it was particularly difficult to find young people who are institutionally involved in nature conservation in their free time. Because of this, we expanded the sample acquisition geographically (in the authors’ home countries of Germany and Austria). This condition may limit the generalizability of the conclusions. The latter would require a representative sample. Another limitation of the present study involves the possibility of participants engaging in multiple groups. We tried to avoid overlaps by also asking all participants about their participation in all three types of organizations (e.g., nature conservation organizations or welfare associations). Only 4.08% of participants demonstrated double engagement, mainly between the socially engaged group and members of nature protection organizations. For participants with double engagement, we can assume a higher tendency toward pro-environmental behavior, as this is driven by prosocial propensity (Otto et al., 2021). Note, however, that such double engagement would have deflated rather than increased the differences between groups in terms of interest in nature, connection with nature and preservation and utilization of it.

## 6. Conclusion

The empirical findings presented in this study suggest that the SIN is a reliable and valid instrument that can be used to measure adolescents’ level of interest in nature as a specific and practically meaningful facet of attitude toward or connectedness to nature. Furthermore, the results of the comparison between members and non-members of environmental organizations indicate a significant difference in the level of interest. Therefore, to promote interest in nature, a stronger engagement with nature should be encouraged (Otto and Pensini, 2017). Additionally, our data suggests a correlation between interest in nature and other environmental attitudes, which may contribute to a deeper understanding of the underlying mechanisms underlying pro-environmental behavior. For example, our scale could help model the interconnections between as well as the prerequisites for environmental attitudes. With the SIN, we provide researchers and educators with an instrument to empirically assess interest in nature as an important component of a more sustainable future. This is of great importance, as the ecological domain seems to be related to the prosocial domain of sustainable development, at least on the individual level (Otto et al., 2021). Prosocial propensity stems from a feeling of connection to a relevant domain. In our case, it is connectedness to nature which serves as a motive for acting on one’s prosocial propensity within the ecological domain and generating further pro-environmental behavior (Otto et al., 2021). Knowing that environmental education interventions foster connectedness to nature as well as pro-environmental behavior, it is important to investigate any recursive effect on prosocial propensity. Only by understanding the interrelatedness of these constructs can we make recommendations on the most effective ESD or environmental education programs (Otto et al., 2021). Here, our scale could supplement even broad existing outcome measures of ESD (e.g., Günther et al., 2022) by contributing a measure of a potential driver of individual sustainable behavior. With the SIN, we can not only investigate the relationship between interest in nature and other environmental variables in more detail, but also find out more about its influence on pro-environmental behavior. It can also be used by practitioners in formal or non-formal ESD settings alike to evaluate their programs, exploring whether they are able to increase adolescents’ interest in nature and thus pave the way for more pro-environmental behavior.

## Data availability statement

The original contributions presented in the study are publicly available. This data can be found at: https://osf.io/w2epc/.

## Ethics statement

Ethical review and approval was not required for the study on human participants in accordance with the local legislation and institutional requirements. Written informed consent to participate in this study was provided by the participants’ legal guardian/next of kin.

## Author contributions

A-LN contributed to the conceptualization, methodology, data collection, validation, formal analysis, writing the original draft, visualization, and project administration. NP contributed to the conceptualization and methodology. SO contributed to the validation, formal analysis, reviewing, and editing the manuscript. AM contributed to the conceptualization, methodology, validation, formal analysis, reviewing, and editing the manuscript, resources, project administration, funding acquisition and supervision. All authors contributed to the article and approved the submitted version.

## Funding

This work was supported by the Open Access Publishing Fund of the University of Vienna (Austria).

## Conflict of interest

The authors declare that the research was conducted in the absence of any commercial or financial relationships that could be construed as a potential conflict of interest.

## Publisher’s note

All claims expressed in this article are solely those of the authors and do not necessarily represent those of their affiliated organizations, or those of the publisher, the editors and the reviewers. Any product that may be evaluated in this article, or claim that may be made by its manufacturer, is not guaranteed or endorsed by the publisher.
