# Supplementary material for: Measuring adolescents’ level of interest in nature: a promising psychological factor facilitating nature protection
Source: Front Psychol. 2023 Jun 21;14:1186557. doi: 10.3389/fpsyg.2023.1186557 (PMC10321522; doi:10.3389/fpsyg.2023.1186557)
Supplement: Supplementary file 1 [file Table_1.pdf]

Supplemental material 1

Supplemental material for Neurohr, A.-L., Pasch, N., Otto, S. & Möller, A. (2023). Measuring adolescents' level of interest in nature: A promising psychological factor facilitating nature protection. Frontiers in Psychology. Vol., 24. doi: 10.3389/fpsyg.2023.1186557

Questionnaire of the validation study

1. 18 items of the Scale of Interest in Nature (SIN)

SIN		Strongly disagree	Disagree	Partially agree	Agree	Strongly agree	Not applicable
3 IN	In my opinion, documentaries and movies on nature are interesting.	☐	☐	☐	☐	☐	☐
5 INi	Plants are boring.	☐	☐	☐	☐	☐	☐
13 INi	I prefer talking about new movies and music, rather than animals.	☐	☐	☐	☐	☐	☐
15 IN	In libraries, I like reading nature books (for example on animals or plants).	☐	☐	☐	☐	☐	☐
16 IN	I would be happy to receive a calendar with nature pictures (for example animals or landscapes) for my birthday.	☐	☐	☐	☐	☐	☐
17 INi	I don't really mind the fact that humans destroy nature.	☐	☐	☐	☐	☐	☐
27 IN	I enjoy discovering nature with my friends more than playing computer games or video games with them.	☐	☐	☐	☐	☐	☐
37 IN	It is exciting to examine bees or other insects with a magnifying glass.	☐	☐	☐	☐	☐	☐
7 INi	I have no personal interest in what happens in nature.	☐	☐	☐	☐	☐	☐
22 INi	If I am being completely honest, I don't care about animals and plants at all.	☐	☐	☐	☐	☐	☐
24 IN	It is important to me to know the names of local animals and plants.	☐	☐	☐	☐	☐	☐
26 IN	Personally, I find it important to know the role of humans in nature.	☐	☐	☐	☐	☐	☐

28 IN	I could imagine collecting feathers, leaves or other things as a hobby.	☐	☐	☐	☐	☐	☐
21 IN	In my spare time I take pictures of flowers, animals and landscapes.	☐	☐	☐	☐	☐	☐
30 IN	In my spare time I participate in projects on preserving nature.	☐	☐	☐	☐	☐	☐
34 IN	In my spare time I examine plants and conduct small experiments with them (e.g., poking them gently, blowing at them)	☐	☐	☐	☐	☐	☐
36 IN	Outside of school I seek out information about animals and plants (for example on the internet or in books).	☐	☐	☐	☐	☐	☐
42 IN	I purposely chose hobbies that allow me to spend a lot of time in nature (e.g., riding, fishing, geocaching).	☐	☐	☐	☐	☐	☐

2. 18 2-MEV items for measuring Preservation (PRE) and Utilization (UTL)

U/P		**Strongly disagree**	**Disagree**	**Partially agree**	**Agree**	**Strongly agree**	**Not applicable**
UTL 1i	For the technical progress some endangerments of the nature must be accepted.	☐	☐	☐	☐	☐	☐
PRE 5	I make sure to save water and electricity.	☐	☐	☐	☐	☐	☐
PRE 7	The state must set aside areas to protect endangered species.	☐	☐	☐	☐	☐	☐
UTL 2i	We need to clear forests in order to grow crops.	☐	☐	☐	☐	☐	☐
UTL 7i	Humans have the right change nature as they see fit.	☐	☐	☐	☐	☐	☐
UTL 9i	Human beings are more important than other creatures (e.g., animals and plants).	☐	☐	☐	☐	☐	☐
UTL 10i	We should remove graden weeds to help beautiful flowers grow.	☐	☐	☐	☐	☐	☐

UTL 6i	Only plants and animals of ecological importance need to be protected.	☐	☐	☐	☐	☐	☐
PRE 1	It upsets me to see countryside taken over by building sites.	☐	☐	☐	☐	☐	☐
UTL 5i	We must build more roads so people can travel to the countryside.	☐	☐	☐	☐	☐	☐
PRE 3	Humankind will die out if we don't live in tune with nature.	☐	☐	☐	☐	☐	☐
PRE 2	I enjoy trips to the countryside.	☐	☐	☐	☐	☐	☐
PRE 4i	Society will continue to solve even the biggest environmental problems.	☐	☐	☐	☐	☐	☐
PRE 8	It is interesting to know what kind of creatures live in ponds or rivers.	☐	☐	☐	☐	☐	☐
PRE 9	Dirty industrial smoke from chimneys makes me angry.	☐	☐	☐	☐	☐	☐
PRE 10i	Our planet has unlimited resources (e.g., oil, wood, coal).	☐	☐	☐	☐	☐	☐
UTL 3i	Nature is always able to restore itself.	☐	☐	☐	☐	☐	☐
UTL 4i	People worry too much about pollution.	☐	☐	☐	☐	☐	☐

Note. Inverse items are marked with an i (for inverted)

3. Inclusion of nature in self (INS)

How interconnected are you with nature? Choose the picture which best describes your relationship to nature.

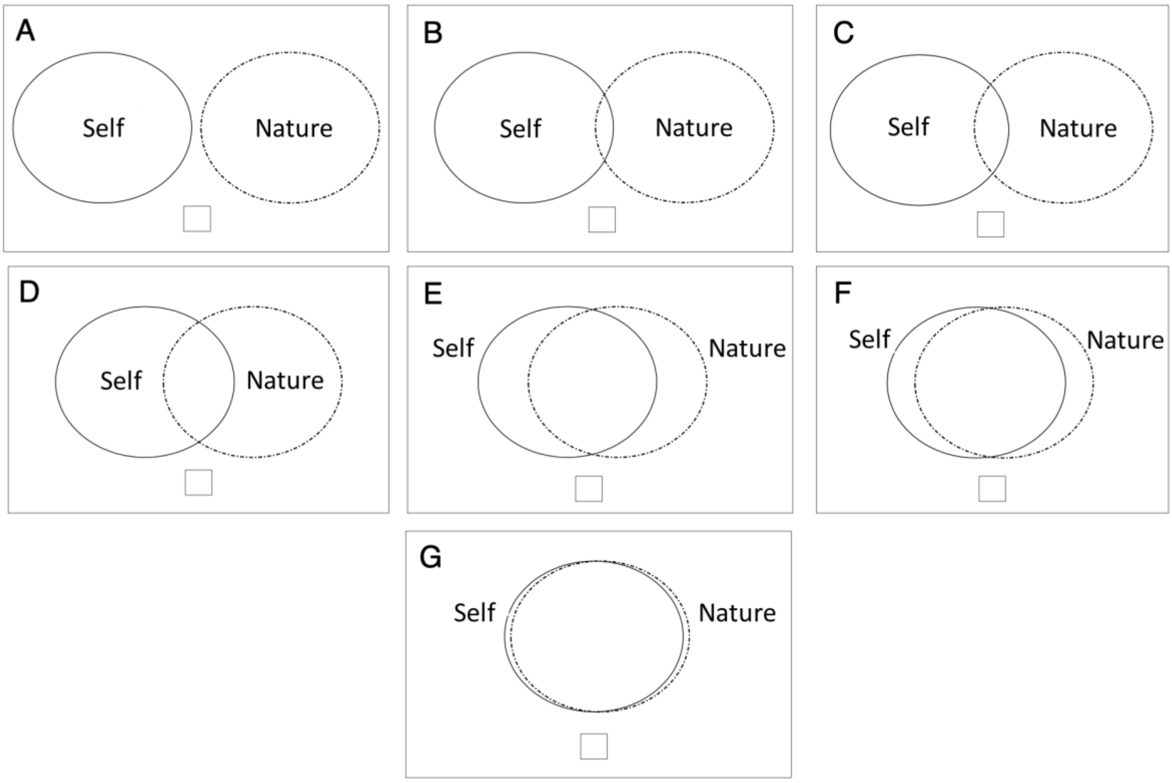

(Adapted from Schultz, 2002)
