# Supplementary material for: Measuring adolescents’ level of interest in nature: a promising psychological factor facilitating nature protection
Source: Front Psychol. 2023 Jun 21;14:1186557. doi: 10.3389/fpsyg.2023.1186557 (PMC10321522; doi:10.3389/fpsyg.2023.1186557)
Supplement: Supplementary file 2 [file Table_2.pdf]

Supplemental material 2

Supplemental material for Neurohr, A.-L., Pasch, N., Otto, S. & Möller, A. (2023). Measuring adolescents' level of interest in nature: A promising psychological factor facilitating nature protection. Frontiers in Psychology. Vol., 24. doi: 10.3389/fpsyg.2023.1186557

Table 1. Original 43 items of the SIN scale (18 final items used in the validation study are in bold)

Item ID	*Item wording*
1 IN	When I see a bird, I know what its species is called.
2 IN	When I look at leaves on trees, I know what species they belong to.
**3 IN**	**In my opinion, documentaries and movies on nature are interesting.**
4 IN	When I go swimming in a lake or the sea, I'm interested in what other species are swimming in the water.
**5 IN**	**Plants are boring.**
6 IN	When I observe insects on flowers, I recognize their species.
**7 IN**	**I have no personal interest in what happens in nature.**
8 IN	Walking through forests or meadows has a positive effect on my mood.
9 IN	When I hear animal voices in the forest, I know which animal they belong to.
10 IN	I enjoy observing animals.
11 IN	I maintain some kind of friendship with an animal (e.g. dog, cat, horse).
12 IN	Playing with animals or petting them gives me joy.
**13 IN**	**I prefer talking about new movies and music, rather than animals.**
14 IN	I do not enjoy taking care of pets.
**15 IN**	**In libraries, I like reading nature books (for example on animals or plants).**
**16 IN**	**I would be happy to receive a calendar with nature pictures (for example animals or landscapes) for my birthday.**
**17 IN**	**I don't really mind about the fact that humans destroy nature.**
18 IN	When I paint animals or plants, it's important to me to draw them as real as possible.
19 IN	In my spare time I like to draw animals, plants or landscapes.
20 IN	I like to collect chestnuts, acorns or leaves (e.g. for crafting, decorating).
**21 IN**	**In my spare time I take pictures of flowers, animals and landscapes.**
**22 IN**	**If I am being completely honest, I don't care about animals and plants at all.**

23 IN	When I travel to a foreign country, it is important for me to get to know animals living there as well.
**24 IN**	**It is important to me to know the names of local animals and plants.**
25 IN	If I could, I would touch exotic animals like snakes or sharks to know how they feel.
**26 IN**	**Personally, I find it important to know the role of humans in nature.**
**27 IN**	**I enjoy discovering nature with my friends more than playing computer games or video games with them.**
**28 IN**	**I could imagine collecting feathers, leaves or other things as a hobby.**
29 IN	When I find mushrooms or berries in the forest, I recognize which ones are edible.
**30 IN**	**In my spare time I participate in projects on preserving nature.**
31 IN	It is important for me to decorate my room with plants or natural materials (e.g. shells, feathers).
32 IN	If possible, I explore what animals live in the water or soil.
33 IN	When I see plants in fields, I recognize what the farmer is growing there.
**34 IN**	**In my spare time I examine plants and conduct small experiments with them (e.g., poking them gently, blowing at them)**
35 IN	I only deal with nature when I have to (e.g. at school).
**36 IN**	**Outside of school I inform myself about animals and plants (for example on the internet or in books).**
**37 IN**	**It is exciting to examine bees or other insects with a magnifying glass.**
38 IN	I enjoy biology classes.
39 IN	If I don't know a leaf or animal I've found, I ask for help at home or from my teachers.
40 IN	In my spare time I catch animals (tadpoles, grasshoppers, frogs) and keep them in a bucket or terrarium.
41 IN	I know a lot about feeding relationships in the forest.
**42 IN**	**I purposely chose hobbies that allow me to spend a lot of time in nature (e.g., riding, fishing, geocaching).**
43 IN	I enjoy looking for animal tracks in the woods with friends or family.
